# Relationship between occlusal force and falls among community-dwelling elderly in Japan: a cross-sectional correlative study

**DOI:** 10.1186/s12877-018-0805-4

**Published:** 2018-05-09

**Authors:** Maki Eto, Shinji Miyauchi

**Affiliations:** 1 Department of Public Health Nursing, Ube Frontier University, Ube, Japan; 2 Division of Linguistics, Department of Health Sciences, Oita University of Nursing and Health Sciences, 2944-9 Megusuno, Oita, 870-1201 Japan

**Keywords:** Community-dwelling elderly, Falls, Occlusal force, Public health nurses

## Abstract

**Background:**

Falls may cause serious health conditions among older population. Fall-related physical factors are thought to be associated with occlusal conditions. However, few studies examined the relationship between occlusal force and falls. To identify the association between occlusal force and falls among community-dwelling elderly individuals in Japan, public health nurses conducted a cross-sectional descriptive study.

**Methods:**

We performed extensive physical assessments of five items: maximum occlusal force, handgrip strength, maximal knee extensor strength, one-leg standing time with eyes open and body sway. We also conducted a questionnaire survey concerning the participants’ demographic characteristics, health status and fall experience during the past year. Mean scores and standard deviations were calculated for age and the total points of the index of activities of daily living. Associations were examined using Mann-Whitney tests and logistic regression.

**Results:**

We examined 159 community-dwelling people aged ≥65 years, who were independent and active, including 38 participants (24.5%) with experience of falls in the past year. Maximum occlusal force had significant correlation with handgrip strength, maximal knee extensor strength, and one-leg standing time and body sway (*P* < .05, respectively). We found weak associations between participants with and without a history of falls in terms of the five physical measurements. Logistic regression analysis showed that fall experience was significantly associated with maximum occlusal force (*P* = 0.004).

**Conclusions:**

This is the first study, led by public health nursing researchers, to examine the associations between maximum occlusal force and falls among community-dwelling elderly in Japan. The results showed that maximum occlusal force was significantly related to the other four extensive physical assessments, and might also suggest that maximum occlusal force assessment by public health nurses could contribute to more sophisticated and precise prediction of fall risks among the community-dwelling elderly. The latest occlusal force measurement device is non-invasive and easy to use. Public health nurses can introduce it at periodical community health checkup assembly events, which might contribute to raising awareness among community-dwelling elderly individuals and public health nurses about fall prevention and prediction.

**Electronic supplementary material:**

The online version of this article (10.1186/s12877-018-0805-4) contains supplementary material, which is available to authorized users.

## Background

Falls are likely to lead to serious or deadly health conditions among older populations in aging societies [1–3]. Major factors related to falls include muscle strength [4, 5] and balance ability [6–8], which have significant associations with occlusal conditions or occlusal force [9]. For example, handgrip force is an upper extremity muscle strength parameter that is significantly associated with occlusal force among older community-dwelling women [10], among the oldest community-dwelling men [11], and among elderly community-dwelling individuals [12]. Okuyama et al. [13] showed that partial or complete lack of teeth among elderly community-dwelling individuals was significantly associated with lower extremity muscle strength. As a balance ability index, one-leg standing time with eyes open is also significantly associated with occlusal force among older populations [10–15]. Yoshida et al. [16] indicated a significant association between the deterioration of occlusal conditions and the number of falls among elderly individuals with dementia.

Public health nurses (PHNs) in Japan have a role in promoting health and preventing diseases in the community by planning, implementing, and evaluating community health programs for the elderly, including fall prevention programs [17, 18]. Regular health examinations for the elderly population are planned through the community health authority and implemented by PHNs. Hirao et al. [10] and Yoshida et al. [16] argued that dental examinations should be included during this regular health check-up for older community- dwelling people. However, to our knowledge, there are few PHN-led studies concerning occlusal force or conditions and few studies related to the association between occlusal force and falls among community-dwelling elderly. Therefore, the aim of this PHN-led study was to examine the associations between maximum occlusal force (MOF) and falls among community-dwelling elderly in Japan and to discuss the possible availability and profitability of occlusal force assessment by PHNs to prevent falls in the community.

## Methods

### Recruitment and sample

We selected a city in southwestern Japan with a population of approximately 170,000 and explained the aim of our study entitled “survey of health conditions and falls among the community-dwelling elderly in a Japanese city” to the health care department of the city office. With support from the department, we recruited volunteer study participants (*N* = 159) who were community residents, 65 years or older, and able to perform activities of daily living (ADL) independently. We described the objectives and procedures of the study to the participants and obtained signed informed consent forms from them.

### Data collection

We used a cross-sectional descriptive correlative approach as the study design. We scheduled study gatherings at nine different community halls in the city from February 17 to March 31, 2014. Each participant chose a convenient time and visited the study site using their own mode of transportation, including walking. There were two measurements, extensive physical assessments of five items including MOF, which took approximately 30 min (Additional file 1), and a questionnaire concerning the demographic characteristics and health status of the participants, which also took approximately 30 min (Additional file 2).

As extensive physical assessment, we measured five items: MOF, handgrip strength, maximal knee extensor strength, one-leg standing time with eyes open and body sway. Moon & Lee [9] described the definition of occlusion as contact between the maxillary and mandibular teeth during chewing or at rest. Therefore, in this study, we defined occlusal force as the force produced by occlusion. MOF (kN) was measured according to a standard procedure using an occlusal force-measuring device (GM10 Morita Occlusal Force Meter; J. Morita Corporation, Tokyo, Japan). Dentures were worn when present. MOF measurement was performed with the participants in an unsupported, natural seated position. We explained the measurement method before it was performed. We did not obtain MOF measurements from participants who reported pain at the beginning of the measurement. We also asked the participants to stop biting as soon as they felt pain or discomfort. MOF was measured unilaterally on the right and left sides at the molar. The maximum value of either the right or the left measurement was used as the MOF value.

Handgrip strength values (kg) of the dominant and non-dominant hands were alternately measured, using Takei Digital Hand Grip Dynamometer T.K.K. 5401 (Takei Scientific Instruments Co., Ltd., Niigata, Japan), during two trials with each patient in an unsupported, natural standing position. The maximum value of either the right or the left measurement was used as the handgrip strength value.

Maximal knee extensor strength (kg) was measured using μTas MF-1 (Anima Co. Ltd., Tokyo, Japan). The participants sat on a chair and held both hands behind their back to support their upper body. The thighs, knees, and ankles were held at a right angle. A sensor pad was attached to the lower front of the shin with an elastic band, and the band was looped around the leg of the chair. We asked the participants to kick their leg with the sensor pad as hard as possible and to keep that strength for five seconds. The trials were conducted twice for the right and left legs. We also asked the participants to breathe out when kicking to reduce the burden on the heart and to stop the trial when they felt pain or discomfort. The maximum value of either the right or the left measurement was used as the knee extensor strength value.

One-leg standing time with eyes open was measured with the hands on the waist while standing on one foot while the other foot was off the floor. The time (seconds) was recorded until the participants lost their balance, began to hop around, moved their hands off the waist, or lowered the raised foot to the floor. They performed one trial for the right foot first; after a 10-s break, they did the same with the left foot. The highest value of either the right or the left measurement was used as the time for one-leg standing, with a maximum score of 120 s.

The total length of body sway was measured using a 5-km/h moving image (cm), Gravi Coda GS3000 (Anima Co., Ltd., Tokyo, Japan), and Eye Trek FMD-150 W (Olympus, Tokyo, Japan). First, the body sway of participants was measured for 30 s in a natural standing position with the eyes open. After 30 s of rest, participants put the Eye Trek gear on their head. As they watched an image moving 5 km/h through the gear, their body sway was measured for 30 s by following a certain point of the body surface. We calculated the total length of the movement of the point as the body sway value. When we performed the body sway measurement, all participants were supported by research assistants standing beside and behind them on the equipment to prevent them from falling. We did not perform the measurement for participants who claimed a tendency for motion sickness, medical history of cerebrovascular disease or epilepsy, or anxiety regarding their health status. Fortunately, we found no such cases.

For the safety of participants and precise data collection, we trained 13 research assistants, including three licensed registered nurses, for one week to skillfully perform the extensive physical assessments. Each assistant was in charge of his/her specific assessment item only. Reproducibility of measurements was examined using peer measurements. We confirmed that the skill levels of the assistants were satisfactory.

We developed our original questionnaire, including previous research regarding health status and falls among the community-dwelling elderly. The questionnaire items were as follows: age; sex; history of disease; denture use; regular/ routine exercise habits such as walking, jogging, or playing sports; ADL using the Tokyo Metropolitan Institute of Gerontology Index of Competence (TMIG-IC) [19, 20]; and falls during the past year. TMIG-IC, which was created in the United States in the 1970s, was modified in the 1980s to assess the competence of Japanese elderly individuals in terms of instrumental self-maintenance, intellectual activity, and social role. The index consists of 13 yes (1 point) and no (0 points) questions. Higher scores (maximum of 13 points) indicated that participants were able to perform ADL better and more independently ([21–23]. TMIG-IC also questions five items regarding quality of life: sense of subjective health, relationship with family, relationship with friends, financial satisfaction, and subjective happiness. Participants were asked to rate their values for the items using a 100-mm visual analogue scale, with the worst value on the left end and the best value on the right.

Before implementing the structured questionnaire, research assistants were trained regarding how to help the elderly answer it. During registration at the survey gatherings, we asked participants their names and postal addresses, so that data could be returned to them after the survey. We distributed a set of data collection sheets including the structured questionnaire with an identification number for each participant. To help the participants answer the questionnaire, the research assistants sat face-to-face with them and read aloud the questions, sentences, and answers slowly. After the participants finished all the measurements and questions, the completed data collection sheets were collected by the research assistants to analyze the data. The full set of the questionnaire sheet included additional question items, which were not relevant to the present study. Therefore, we did not provide the data of those question items in this paper.

### Data analysis

We obtained descriptive statistics regarding age, sex, history of disease, current disease status, denture use, presence or absence of regular/routine exercise habits or playing sports, ADL using TMIG-IC, and falls during the past year. Mean scores and standard deviations were calculated for age and the total points of the TMIG-IC. Associations were examined using Mann-Whitney tests for falls and MOF, handgrip strength, maximal knee extensor strength, one-leg standing time with eyes open, and total length of body sway using a 5-km/h moving image. We performed logistic regression to determine odds ratios (ORs) of falls. The covariates included the following five items which have significant correlation and are related with muscle: MOF, handgrip strength, maximal knee extensor strength, one-leg standing time with eyes open, and total length of body sway with a 5-km/h moving image. We performed statistical calculations using SPSS version 22.0 for Windows (IBM Japan, Tokyo, Japan) with a significance level of *P* < 0.05.

### Ethical considerations

All participants submitted consent forms to the study researchers. We fully disclosed the purposes and significance of the study both orally and in written form. We assured the participants that participation was voluntary and that they could stop their participation at any time without any disadvantages. It was ensured that we would keep the data anonymous and confidential and would limit data use only to the study. All participants, research staff, and measurement instruments were insured against accidents. The study was formally approved by the institutional review board of the author’s university.

## Results

### Characteristics of participants

We examined a total of 159 participants; 56 were male (35.2%) and 103 were female (64.8%). The average age was 75.7 ± 6.8 years for men; it was 73.5 ± 6.1 years for women. Ninety participants (56.6%) had a history of diseases and 131 (82.4%) had current diseases. Ninety-eight people (61.6%) performed regular physical exercise. Male participants had an average TMIG-IC score of 12.25 ± 1.16 and female participants had an average TMIG-IC score of 12.20 ± 1.19 on a scale of 0 to 13. Of these participants, 38 (24.5%) experienced falls in the past year. Sixty-five participants (40.9%) wore dentures.

### Extensive physical assessments and falls

The average strength values of MOF with and without falls were 0.35 ± 0.23 kN and 0.25 ± 0.20 kN, respectively. The average strength values for handgrip of the participants with and without falls were 27.50 ± 7.87 kg and 30.41 ± 9.26 kg, respectively. For maximal knee extensor strength, the average scores of the participants with and without falls were 13.87 ± 7.28 kg and 11.70 ± 5.40 kg, respectively. In terms of body balance, the average one-leg standing times with eyes open for those with and without falls were 46.47 ± 50.82 s and 62.58 ± 79.62 s, respectively. For total length of body sway, the average scores for those with and without falls were 42.04 ± 16.32 cm and 50.05 ± 19.04 cm, respectively. Regarding the five items of physical assessment, MOF had significant correlation with handgrip strength, maximal knee extensor strength, and one-leg standing time (*P* < .01, respectively), and with body sway (*P* < .05) (Table 1). We found weak associations between participants with and without a history of falls (Table 2).

**Table 1:** Correlative analysis among extensive physical assessments

	Occlusal force	Handgrip strength	Knee extensor strength	One-leg standing with open eyes	5 km/h-image body sway: total length	N	M	SD
Occlusal force	―					155	.27	.21
Handgrip strength	.382 **	―				157	28.79	8.55
Knee extensor strength	.263 **	.457 **	―			157	11.63	5.68
One-leg standing with open eyes	.369 **	.129 *	.202 *	―		157	55.38	71.59
5 km/h-image body sway: total length	−.196 *	.070 *	−.096	−.291 **	―	157	47.72	17.63

Pearson product-moment correlation coefficient, **: *P* < 0.01, *: *P* < 0.05. M stands for mean. SD stands for standard deviation

**Table 2:** Extensive physical assessments: with or without experience of falls

With experience (*n* = 38)	Without experience (*n* = 121)	*P*-value
Maximum occlusal force, mean kN ± SD		
0.35 ± 0.23	0.25 ± 0.20	.051
Handgrip strength, mean kg ± SD		
27.50 ± 7.87	30.41 ± 9.26	.156
Knee extensor strength, mean kg ± SD		
13.87 ± 7.28	11.70 ± 5.40	.068
One-leg standing, mean sec ± SD		
46.47 ± 50.82	62.58 ± 79.62	.101
5 km/h-image body sway: total length, mean cm ± SD		
42.04 ± 16.32	50.05 ± 19.04	.062

Mann-Whitney U test, SD; standard deviation

### Associations of falls with measurement items

Logistic regression analysis showed that fall experience was significantly associated with MOF (OR, 0.49; 95% confidence interval [CI]: 0.09–2.62; *P* = 0.004), handgrip strength (OR, 0.39; 95% CI, 0.17–0.90; *P* = 0.027), maximal knee extensor strength (OR, 2.04; 95% CI, 1.17–3.55; *P* = 0.012), and one-leg standing time (OR, 8.40; 95% CI, 1.10–64.26; *P* = 0.040). In this study, there was no significant association between falls and the average total length of body sway with a 5-km/h moving image (OR, 3.44; 95% CI, 0.38–31.53; *P* = 0.072) (Table 3). The values of C statistic concerning the five items are as follows: MOF, 0.648; handgrip strength, 0.578; maximal knee extensor strength, 0.608; one-leg standing time, 0.533; and the average total length of body sway with a 5-km/h moving image, 0.654.

**Table 3:** Odds ratio between fall experience and extensive physical assessments (*n* = 159)

Measures	Mean	Odds ratio	95% CI		*P*-value
			low	upper	
Maximum occlusal force	0.27	.49	.09	2.62	.004*
Handgrip strength	28.69	.39	.17	.90	.027*
Knee extensor strength	11.62	2.04	1.17	3.55	.012*
One-leg standing with open eyes	55.40	8.40	1.10	64.26	.040*
5 km/h-image body sway: total length	52.11	3.44	.38	31.53	.072*

Logistic regression, *P < 0.05. CI stands for confidence interval

## Discussion

This study sought to examine the associations between extensive physical assessments including MOF and fall experience among elderly community-dwelling individuals. High scores on the TMIG-IC in terms of the quality of ADL seemed to indicate that participants were healthy and active, and had similar physical and functional characteristics.

The result of our study showed that MOF was significantly related to the other four extensive physical assessments. With regard to hand-grip strength, lower extremity strength and one-leg standing time, the result was similar to that in the previous study [15]. We identified significant tendencies between the participants with fall experience and those without it in terms of all the extensive physical assessments. However, logistic regression analysis revealed that MOF had the second strongest association with falls, after knee extensor strength. The results of this study not only supports the argument in the previous study [14] that examining MOF is appropriate for determining the functional abilities of the lower extremities and body balance, but might also suggest that MOF assessment itself could contribute to predicting fall risks among the community-dwelling elderly. Abe et al. [24] also contended that the assessment of fall risks could be more sophisticated and exact by combining examinations of lower extremity muscle strength and balance ability. Our study suggests that adding MOF assessment to the examinations of lower extremity strength and balance ability could make the potential of fall risk prediction much more sophisticated and precise.

We found that the MOF average score among participants with fall experience was higher than that of participants without falls: 0.35 ± 0.23 kN and 0.25 ± 0.20 kN, respectively. This might be because participants with a partial lack of teeth put more pressure on the remaining teeth than do participants with all their teeth. Song-Yu et al. [8] reported that elderly individuals with a partial lack of teeth had significantly less stable posture. Proper clenching excites the human motor system and affects human postural control [25]. In this study, we did not examine the relationship between MOF and denture use or between MOF and the number of remaining teeth. However, due to the higher MOF scores among participants with fall experience, we suggest that they have unfavorable dental conditions that might have affected their balance ability [13], resulting in falls. It is also implied that bite adjustment or denture use may improve postural control.

The device used to measure occlusal force in this study, GM10, is a portable tool that is easy to use and inexpensive. It is also less invasive physically and psychologically for those who undergo MOF measurements [12]. It can obtain objective data instead of subjective (and sometimes false) individual perceptions of MOF [26]. By using this handy tool, PHNs can easily assess MOF among elderly community-dwelling individuals. PHNs can collect data during regular community health check-ups, share and analyze the data, and recognize the tendencies of MOF conditions among the community-dwelling elderly. This may lead PHNs to provide individual advice to each of the elderly individuals and to raise awareness on oral health among them [16], which could potentially help to prevent future falls and reduce health care expenditures. PHNs could also share up-to-date information about risk of falls in the community with other healthcare professionals. Therefore, PHNs should check the MOF of the elderly to raise awareness on fall prevention.

One of the potential confounders on the association between occlusal force and falls is bone calcium. A systematic literature review discusses usefulness of bone density measurement in fallers in France [27]. Another review shows the evidence for the value of calcium supplementation for healthy musculoskeletal ageing [28]. However, the present study focused on extensive physical functions, which PHNs might be able to instruct directly to the community-dwelling elderly for healthcare. Thus, we did not discuss bone calcium as a confounder on the association.

### Study limitations

This study had several limitations. First, the sample was small and limited to a population of only one city in Japan, which means that elderly individuals living in different areas could have had different experiences. Besides, the demographic characteristics of the sample do not seem to reflect those of the whole Japanese elderly population in terms of age, sex and comorbidity. Thus, it is not appropriate to generalize the results of the present study. Second, the study participants were relatively active community-dwelling elderly. Therefore, generalization of the present discussion cannot be applied to vulnerable and frail populations in the community, such as those with lower levels of ADL. Third, this study used a cross-sectional survey approach, using sample extracted from a limited area of a particular city in Japan. This means that the study design cannot refer to the cause and effect between MOF and falls. A prospective follow-up cohort study should be conducted to determine the cause-effect relationship between changes in MOF and falls.

## Conclusions

This is the first study conducted by public health nursing researchers, which examined the associations between MOF and falls among community-dwelling elderly in Japan. The results showed that MOF was significantly related to the other four extensive physical assessments, and might also suggest that MOF assessment by public health nurses could contribute to more sophisticated and precise prediction of fall risks among the community-dwelling elderly. The latest occlusal force measurement device is non-invasive and easy to use. Public health nurses can introduce it at periodical community health checkup assembly events, which might contribute to raising awareness among community-dwelling elderly individuals and public healthcare providers about fall prevention and prediction.

## Additional files

Additional file 1: Physical assessment data sheet. (DOCX 17 kb)

Additional file 2: Questionnaire. (DOCX 20 kb)

## Abbreviations

ADL: Activities of daily living
CI: Confidence interval
M: Mean
MOF: Maximum occlusal force
ORs: Odds ratios
PHNs: Public health nurses
SD: Standard deviation
TMIG-IC: Tokyo Metropolitan Institute of Gerontology Index of Competence
