# Supplementary material for: Relationship between occlusal force and falls among community-dwelling elderly in Japan: a cross-sectional correlative study
Source: BMC Geriatr. 2018 May 9;18:111. doi: 10.1186/s12877-018-0805-4 (PMC5944160; doi:10.1186/s12877-018-0805-4)
Supplement: Supplementary file 1 — Physical assessment data sheet. (DOCX 17 kb) [file 12877_2018_805_MOESM1_ESM.docx]

Additional file 1: Physical assessment data sheet

ID no.

Date of birth: (Month, date, year)

Age:

Sex: Male/Female

Date of measurement: (Month, date, year)

Height: cm

Weight: kg

Handgrip strength: Right kg; Left kg

Maximal knee extensor strength: Right kg; Left kg

Maximal occlusal force: kN

One-leg standing time with eyes open: seconds

Total length of body sway with a 5 km/h moving image: cm
