# Supplementary material for: Relationship between occlusal force and falls among community-dwelling elderly in Japan: a cross-sectional correlative study
Source: BMC Geriatr. 2018 May 9;18:111. doi: 10.1186/s12877-018-0805-4 (PMC5944160; doi:10.1186/s12877-018-0805-4)
Supplement: Supplementary file 2 — Questionnaire. (DOCX 20 kb) [file 12877_2018_805_MOESM2_ESM.docx]

Additional file 2: Questionnaire

ID no.

Date of birth: (Month, date, year)

Age:

Sex: Male/Female

1. How many housemates do you have?

2. Are you currently being treated for any medical condition(s)? Yes/No

If you answered “yes,” please specify the name of the condition(s).

3. Have you ever had, or currently have, any of the below medical conditions? Yes/No

1. Cerebral stroke 2. Heart disease 3. Hypertension 4. Diabetes

5. Cataract 6. Parkinson’s disease 7. Osteoporosis 8. Osteoarthritis of the knee

4. Are you currently using dentures? Yes/No

5. How long have you been using dentures?

1. Less than a year

2. 1-2 years

3. 2-5 years

4. 5-10 years

5. 10 years or more

6. Do you wear your dentures every day? Yes/No

7. Do you exercise? Yes / No

If you answered “yes”, please choose the form of exercise you do the most.

1. Walking 2. Radio gymnastics 3. Cycling 4. Gate ball (Japanese croquet)

5. Hiking 6. Dancing 7. Jogging 8. Weight training 9. Martial arts

10. Swimming 11. Ball sports

12. Others (Please specify: )

8. How long have you been doing this form of exercise?

For years, and/or months

9. How often do you do this form of exercise?

times/week, times/month, times /year

10. How many minutes do you spend on this form of exercise? minutes

11. Please answer the following questions about your daily activities.

1. Can you go out by yourself on a bus or train? Yes/No

2. Can you shop for your daily necessities? Yes/No

3. Can you prepare your meals by yourself? Yes/No

4. Can you perform the procedures to pay your bills? Yes/No

5. Can you withdraw or put in deposits/savings at a bank/post office? Yes/No

6. Can you fill in your pension-related documents? Yes/No

7. Do you read the newspaper? Yes/No

8. Do you read books/magazines? Yes/No

9. Are you interested in health-related articles and programs? Yes/No

10. Do you visit your friends’ houses? Yes/No

11. Do you give advice to family and/or friends? Yes/No

12. Can you use public transport and visit a sick person? Yes/No

13. Do you talk people younger than you? Yes/No

Thank you for your answers.

End of questionnaire
